# Diabetic foot disease in the United Kingdom: about time to put feet first

**DOI:** 10.1186/1757-1146-5-26

**Published:** 2012-10-11

**Authors:** Alistair D McInnes

**Affiliations:** 1 School of Health Professions, University of Brighton, Eastbourne, UK

## Abstract

Diabetes is now the biggest cause of amputation, stroke, blindness and end stage renal failure. It causes many deaths from cardiovascular disease. Foot ulcers and amputations reduce the quality of life, increase mortality and involve lengthy stay in hospital. Many people who have an ulcer eventually require surgery. The economic cost to the nation is spiralling out of control with estimates of 10% of the entire NHS budget spent on diabetes. This paper aims to explore the burden of diabetic complications and how policy, guidelines and audit highlight the discrepancies in the quality of diabetes care with particular reference to diabetes foot services. The findings suggest that the NICE guidelines for diabetes foot care are not being adhered to and that the variation in preventative amputations across England is unacceptable. Diabetes UK, the national charity for diabetes is leading a campaign to improve diabetic foot care in light of the available published health information.

## Background

Diabetes is one of the most challenging health problems in the 21^st^ century [1]. The recent Association of Public Health Observatories prevalence model suggests that there are 3.1 million people with diabetes in England and if this trend continues, the number will rise to an estimated 4.6 million of the adult population by 2030 [2]. This increase in prevalence has significant economic importance to say nothing of the associated burden of associated mortality and morbidity [3]. The prevalence of diabetes is approximately four times higher than all cancers and continues to rise [4].

There are two primary forms of diabetes, Type 1, characterised by the absence of insulin and Type 2 characterised by insulin resistance. In the UK, approximately 10% have Type 1 and 90% have Type 2 [5]. Every year, the condition is associated with 75,000 deaths, many of which are preventable [6]. The associated tissue complications can be devastating, these include blindness, kidney failure, cardiovascular and cerebrovascular disease and amputation [5]. Approximately 6000 people with diabetes have a leg,foot or toe amputation each year in England and If current trends continue, the amputation rate will increase from 6000 in 2009/10 to more than 7000 in 2014/15 [7].

The cost of diabetes care to the NHS was almost £10 billion in 2011, which is 10% of the total NHS budget. Most of the spending was on managing avoidable complications, accounting for 80% of the total NHS diabetes cost [8]. When indirect costs are added to the direct costs described above for 2010/2011, the figure rises to £23.7 billion with projected estimates for 2035/2036 of £39.8 billion (direct plus indirect costs) [8]. Indirect costs are estimated from mortality data, sickness data, potential loss of productivity among people who remain in work, and informal care [8]. All of the aforementioned data relates to the UK. However, for this paper, the majority of data is based on England and Wales only, unless otherwise stated.

One of the most costly consequences of diabetes in England are significant hospital admissions, given that at any one time around 15% of inpatients have been recorded as having diabetes [9]. The length of stay tends to be longer for those with diabetes with a recorded 4,892,000 nights spent in hospital during 2009-10 which is 19.4% more than for patients without diabetes [9]. Life expectancy is reduced for people with diabetes from 6 to 20 years [10] and deaths from diabetes in England are high with 26,300 [between ages 20-79] in 2005 with an anticipated increase as diabetes prevalence rates rise [11]. In the UK, diabetes is the main cause of blindness in people of working age [12,13], increased rates of kidney failure, increase in amputations and is a main contributor to cardiovascular disease [14].

The UK Government recognises the impact that diabetes has on the population and outlined a vision for diabetes care in England in the *National Service Framework for Diabetes[NSF]* published in 2001 [15]. The Framework states twelve standards of care to be achieved by 2013. However, there is still much to be achieved despite some significant improvements across the country [16]. With the increased prevalence in diabetes and other chronic conditions associated with an unhealthy lifestyle, the Department of Health responded with reforms to improve the health of the nation and improve the quality of health care. The introduction of the *NHS Outcomes Framework* 2012/13 sets out national outcomes that the NHS should be aiming to achieve. These include preventing people from dying prematurely and enhancing quality of life for people with long term conditions [17], which clearly includes those with diabetes. Other Department of Health policy documents that influence diabetes care include the white paper *Equity and excellence: Liberating the NHS 2010* and the *Health and Social Care Bill 2011*. One of the key organisations that are funded by the Department of Health is *NHS Diabetes.* This organisation has also supported the development of the *National Diabetes Information Service* which also includes the *National Diabetes Audit.* This organisation is the catalyst to achieve the standards set out in the Diabetes Framework and in the National Outcomes Framework and they have developed specific programmes to improve the quality of diabetes care across England.

NHS Diabetes have several networks including a national footcare network. A key objective is to reduce the rate of amputations by 50% over a period of five years [18]. Working alongside NHS Diabetes is the *National Institute for Clinical Excellence* [NICE]. NICE is funded by the Department of Health and have a number of functions including the provision of detailed evidenced based guidelines and quality standards for all aspects of diabetes care. In addition, NICE has helped to improve the *Quality and Outcomes Framework [QOF]* in 2010/11, by recommending new indicators to help improve the care of patients with conditions such as diabetes, dementia and mental health problems. The QOF framework is a voluntary programme for GPs to provide evidence of achievement that certain clinical and health indicators have been achieved. This scheme also provides valuable data about diabetes care.

*The NHS Health Check* programme which was announced in 2008 is a systematic programme for people aged between 40-74 to assess people’s risk of developing Type 2 Diabetes, heart disease, stroke and kidney disease [19]. However in 2011 only half of the health checks have been offered [20]. Whilst the NSF for Diabetes and other organisations including the valuable NHS Diabetes have contributed significantly, the recent publication of the *State of the Nation 2012 England*[20] highlights the alarming trend in the increase of complications. From 2006 to 2010, retinopathy increased by 118%, stroke by 87%, kidney failure by 56%, cardiac failure by 43%, angina by 33% and amputations by 26% [20].

The picture regarding amputation varies significantly across England. Recent data has shown that both major and minor amputations in people with diabetes varies tenfold across all of the Primary Care Trusts (PCT) in England. Over a 3 year period to March 2010, there were 16,693 amputations in people with diabetes [21]. This variation in amputation rate suggests that diabetes foot care is inadequate in many parts of the country. However the overall amputation rate increase is considerably less than for the other tissue complications previously described.

This paper aims to consider the costs and impact of the complications of diabetes, and whether there is greater focus on any one complication. Diabetic foot disease has major consequences in terms of morbidity and associated mortality and diabetes foot services may fall short of Government expectations.

### Epidemiology, impact and costs of diabetes in the UK

Successive governments in the UK have attempted to improve the NHS by a plethora of white papers from the Department of Health and more recently through the Health and Social Care Bill 2012. The bill has been regarded as the most controversial to date, with some political commentators suggesting that there is a “privatisation by stealth” agenda in order to decrease the spiralling increase in costs of the NHS. Others suggest the Bill offers greater patient choice and influence over service provision. The current budget of the NHS is around £106 billion of which almost £10 billion is spent on diabetes care [8].

It is timely to consider the comparative costs of diabetes and its complications, given that there is data now available via the National Diabetes Information Service, the National Diabetes Audit and many research publications and economic reports. Given the variation in amputation rate across England [21] and the estimated annual expenditure (2010-11) on foot ulcers and amputation to be between £639,015,210 and £661,767,953, it is important to explore the costs of providing foot care and to identify examples where service improvements have been made in both provision of care and cost savings. The prevalence rates of diabetes complications from 2006-2010 are displayed in Table 1. 

**Table 1:** Prevalence rates of diabetic complications (2006-2010)

Retinopathy	increased by 118%
Stroke	increased by 87%
Kidney failure	increased by 56%
Cardiac failure	increased by 43%
Angina	increased by 43%
Amputations	increased by 26%

### Epidemiology of diabetic complications

There are 2.9 million people with diabetes in the UK [22] and diabetes is responsible for more than one in ten (11.6%) [23] deaths in England in the 20-79 years old group and is the fifth most common cause of mortality in the world [24]. Half of all deaths from diabetes are due to cardiovascular disease and stroke [23]. Cardiovascular disease accounts for 44% of fatalities in people with Type 1 diabetes and 52% in Type 2 [14] and people with Type 2 have a two-fold increased risk of stroke within the first five years of diagnosis [25]. Kidney disease accounts for 21% of deaths in Type 1 and 11% in Type 2 diabetes [14].

Diabetes is the leading cause of blindness in people of working age in the UK [20] and there are 4,200 people in England who are blind as a result of diabetic retinopathy. This number increases by 1,280 each year [26]. Diabetes carries an increased risk of a person undergoing lower extremity amputation 23 times higher than of a person without diabetes [21]. Key data from a report from Holman et al. [21] identified 34,109 amputations over a three year period in England, of which 16,693 (48.9%) were in people with diabetes. The amputation rates in England for people with diabetes varies ten-fold - both major (range 0.22-2.20 per 1000 person-years) and minor (range 0.30-3.25 per 1000 person-years) [21].

### Hospital impact of diabetic complications

Data from the National Diabetes Audit 2011 identified that people with diabetes account for 19% of all hospital inpatients at any one time [27]. The most common reason for admission overall is foot disease, however for Type 1 patients, ketoacidosis predominates. The length of stay for inpatients with diabetes tends to be longer than those without diabetes, with a median stay of 8 nights compared with 5 nights. This results in about 5,912,836 bed days per year [28].

Of the patients that had been admitted for the management of diabetes, the highest proportion (47%) were admitted with active foot disease. See Table 2 for other reasons for admission. Hospital Episode Statistics record 72,459 inpatient episodes for diabetes and foot ulcer or amputation for 2010-11 [28]. Diabetic patients may be admitted to hospital for reasons other than for foot ulcer or may develop a foot ulcer during their stay. An analysis by Kerr [2] in 2012 estimated that in 34,836 admissions coded to non-foot- ulcer related Healthcare Resource Group (HRG), there were 417,804 excess bed days for patients with foot ulceration. An HRG is a grouping of patient events that consume a known resource. This is based on the diagnosis and anticipated intervention procedure. Excess bed days are calculated based upon the expected number of days that a procedure would normally take and the number that are actually used. 

**Table 2:** Reasons for admission to hospital (National Diabetes Inpatients Audit of 2011)

**Active foot disease**	**47% (of 9% of diabetic patient admitted specifically for the management of diabetes)**
Hypoglycaemia	16.2%
Hyperglycaemia	17.9%
Ketoacidosis	13.5%
Hyperglycaemic hyperosmolar state	5.4%

*Important to note that 66.6% were admitted for other medical reasons and 24.4% for non-medical/surgical reasons.

*Total number of 11,866 inpatients with diabetes.

### Cost of acute diabetes complications in the UK

The total cost of direct patient care in the UK in 2010/11 is estimated at £9.8bn which is approximately 10% of the NHS budget [8]. The cost of complications with those with either Type 1 or Type 2 is estimated at £7.7bn [8]. Table 3 displays the estimated costs for diabetic complications observed by Hex and colleagues [8]. From their health economics paper, they demonstrate that foot ulcer and amputation costs for 2010/11 were the most expensive complication to treat in terms of hospital costs, followed by kidney failure and other renal costs. However it is evident that if all cardiovascular events were considered in combination, they would be the most expensive of all. 

**Table 3:** Estimated costs for diabetic complications (2010-2011)

Foot ulcers and amputations	£985,600,282
Kidney failure	£514,066,538
Ischaemic heart disease	£509,656,332
Myocardial Infarction	£603,069,221
Stroke	£287,931,944

### Best practice management of diabetes complications

The Department of Health set out standards of care for diabetes in 2001 in the *National Service Framework for Diabetes* and these standards have been reinforced by NICE in 2011. The Department aims were to improve health outcomes, raise the quality of diabetes services and reduce variations between them [15]. In addition the Department have improved the identification of people with diabetes via the GP practice registers and the *Quality and Outcomes Framework (QOF)*. The QOF allows for the development of clinical and health improvement indicators across a range of areas including diabetes. NICE are instrumental in the development of these indicators. It is difficult to identify precise spending on Diabetes in the NHS in England and the National Audit Office estimated that at least £3.9 billion was spent in 2010, approximately 4% of the entire NHS budget. The Audit Office acknowledges that this could be an underestimation [29].

### Aims not achieved

The Department of Health facilitated the development of a national clinical audit with the Healthcare Quality Improvement Partnership and there is evidence that the standards are not being achieved across the country. In 2009-10, only half of the people with diabetes received all of the recommended care processes to reduce the risk of future complications [20]. Furthermore less than one in five people with diabetes are achieving treatment standards that reduce the risk of developing diabetes related complications [29]. There is a great variation across the country, with the percentage of people with diabetes receiving all nine recommended care processes ranging from 6 per cent to 69 per cent between primary care trusts [20]. In terms of achieving treatment standards, the alarming figures from the National Diabetes Audit for the year 2009-10, demonstrated that 84% of patients did not achieve desired standards for blood glucose, blood pressure and cholesterol which clearly puts them at risk of developing avoidable complications in later life [29]. One of the nine basic care processes for people with diabetes to be delivered on an annual basis includes a foot examination. Evidence from the National Diabetes Audit for 2010-11 identified that 84.4% of people with diabetes received a foot examination which is an improvement of +0.26% from the previous year [29]. However, that suggests that 15.6% did not receive an annual foot check and when consideration is given to the large figures involved, suggests that percentage equate to over 200,000 people. Given that diabetes foot complications are a considerable cost to the NHS, the annual foot care examination data is disappointing.

### Potential cost savings

It is difficult to identify the spending on diabetes care in NHS England. Spending on diabetes has increased from £0.9 billion in 2006-07 to £1.3 billion in 2009-10. The Audit Office recognise that this figure is probably an underestimate as there is a lack of accurate cost data for primary care and community services and that hospital cost mechanisms can be rather opaque. The Audit office has calculated that £170 million a year could be saved by the NHS with improved management of diabetes care. The estimated savings could come from reduced hospital admissions and emergency readmissions, reduced insulin errors in hospital and a reduction in late referrals to specialist foot teams. It has been considered that a reduction of 50 per cent late referrals could save £34 million a year by a reduction in amputations [29].

### Future funding of diabetes complication management

One of the initiatives from the Department of Health was the development of a national screening programme for diabetic retinopathy [29]. The programme aims were to reduce the prevalence of blindness through early detection and treatment of diabetic retinopathy. The programme was supported by the DOH funding the purchase of digital cameras and other equipment for retinal screening. There were 91 programmes implemented between 2003-08. In 2010-11, 79 per cent of people with diabetes had been screed for retinopathy [29].The programme has not been fully assessed as there is a lack of data on those screened who have required follow up treatment or have been registered blind [29].

Primary Care Trust spending on diabetes complications falls under programme budgeting data as part of their spending on 23 programmes of care. This data should inform commissioning decisions about best practice spending on diabetes care. However, as previously mentioned there is a lack of quality cost information for primary care and community services where the majority of diabetes care is provided. There is a wide variation of recorded diabetes related spending between primary and secondary care across primary care trusts.

### NHS Diabetes

The development of NHS Diabetes and the appointment of a national clinical director for diabetes have led to significant improvements in diabetes services. The aim of NHS Diabetes is to provide a link between diabetes strategy and frontline improvements for patients [30]. The core of their work is to aid the implementation of the National Outcomes Framework and NICE Quality Standards for Diabetes. There are a number of spokes to the hub of NHS Diabetes in the form of networks which include paediatric, older people, inpatient, foot care, pregnancy and insulin pump networks.

One of the main targets of the foot care network is to reduce the amputation rate by 50% by 2018. Other goals endorse the NICE Quality standards and objectives of the National Outcomes Framework that relate to diabetes foot disease in terms of standards of service and inequalities and variation in healthcare. The NHS however is about to undergo a seismic change as a result of the *Health and Social Care Act 2012.* It is beyond the scope of this paper to capture all of the main aspects of the Act, suffice to state that at the core of the Act is; a focus on putting clinicians at the heart of commissioning, the facilitation of innovation, empowerment of patients and the drive for improvement in public health. This focus is captured in the *National Outcomes Framework 2011-12* which features five domains with aspirational aims which include: (i) preventing people from dying prematurely; (ii) enhancing quality of life for people with long term conditions; (iii) helping people to recover from episodes of ill health or following injury; (iv) ensuring that people have a positive experience of care and (v) treating and caring for people in a safe environment and protecting them from avoiding harm. NICE quality standards featuring diabetes are referred to in the first two domains described.

## Discussion

The new organisational structure of the NHS will include a National Commissioning Board, accountable to the Secretary of State for Health, that will guide and support clinical commissioning groups who directly commission services for their populations [31]. There are numerous documents that have been reviewed to inform this paper and the complexities of funding mechanisms, commissioning, costings, interpretation of national audits and other diabetes information products including economic analyses are most challenging to unravel. The fact remains that diabetes foot disease accounts for more hospital bed days that any other complication, impacts on the quality of life, results in significant morbidity and subsequently increased mortality [28], it has been described as a Cinderella service and there is evidence to support such a claim. However, conversely, there is a significant health spend on diabetic foot disease. Expenditure on foot ulcers is around 0.6% to 0.7% of all NHS spending and approximately £1 in every £150 the NHS in England spends each year is on diabetic foot ulcers [32]. This equates to £324,761,157 spent in Primary, Community, Outpatients and Accident and Emergency on diabetic foot ulcers and £256,698,817 spent on inpatients with foot ulcers and amputations [28].

It is evident from the National Service Framework for Diabetes, the National Outcomes Framework, NHS Diabetes, Diabetes NHS strategy, NICE Quality Standards for Diabetes and Diabetes UK 15 health care essentials, that the Department of Health and the national charity, Diabetes UK share goals and targets for diabetes care and complications that do not single out any one complication more than another. However it is evident from the report on the management of adult services in the NHS by the National Audit Office [29] that the implementation of all of the quality standards that have been set for diabetes care have not been met and furthermore, there is a significant variation in the standards of care across all of the primary care trusts in England. Before identifying all of the reasons for failure to meet the standards, it may be useful to consider the mechanisms of provider payment as this may be a contributing factor to the discrepancies identified.

### Payment by Results (PbR)

The Payment by Results is a tariff based system that is used by commissioners to pay the providers of healthcare. There are nationally determined currencies and set prices for the tariff. The unit of healthcare may be an outpatient attendance, a stay in hospital or a year of care for a long term condition. The currencies are in the form of a health resource group which captures both a diagnosis and subsequent intervention which are individually coded accordingly. The healthcare resource group also includes the timeframe, e.g. from hospital admission to discharge. An example of on HRG and mean tariff in relation to diabetic foot disease is: HRG: *Amputation without major complications* with a mean tariff of £9297 and an HRG: *Amputation with major complications* with a mean tariff of £14,960 [28].

### Standard contract

A standard contract in the NHS is between the provider and the commissioner which is legally binding and is based on the payment by results and nationally agreed tariffs. The Health and Social Care Bill will lead to some changes to the standard contract for 2012/13. There is a greater emphasis on the services being commissioned. The contract will bring together the core services namely acute hospital, ambulance, community and mental health/learning disability services. The contract also includes indicators for delivery of high quality services. Service specifications can be in a number of different formats including care pathways. This type of service specification may be preferable for diabetes foot services.

### Commissioning for quality and Innovation (CQIN)

All contracts are required to include the national incentive payment framework scheme. The CQIN scheme allows the provider to identify a service for ambitious improvement and innovation to enhance the quality of the service. Successful CQIN activity carries a financial incentive to the provider organisation.

### Quality and Outcomes Framework (QOF)

This framework has previously been described in this paper and provides a financial incentive for general medical practices to achieve a series of targets. There are several standards in relation to diabetes and also include standards for foot screening and risk assessment [33].

To attempt to unravel the complexities of funding and attempt to identify priorities in diabetes care is a complex process. The National Audit Office concluded that the variations in diabetes care cannot be explained by need or spending alone [28]. They may be as a result of differences in local organisation and management of the service. The Audit Office also identified that the NHS lacks clarity on the most effective ways to deliver diabetes services and that there is variation in patient education, diabetes training and in the provision of diabetes specialist nurses [28].

### Podiatry services

The availability of podiatry services for inpatients with diabetic foot problems is a major issue. The National Inpatients Diabetes Audit (2011) included 212 hospitals and 136 Trust sites. The report considered the provision of Podiatry to be poor with an average of 5.0 hours per week per 300 hospital beds spent on inpatient care. This is of particular concern considering that 11.6% of admissions have a history of foot disease and foot disease accounting for nearly half of the diabetes specific admissions. Further disappointing data shows that there has been an increase from 26.8% to 30.9% in the number of sites with no podiatry provision.

Clearly, despite the intentions of all of the national guidance on diabetes care, podiatry services and the poor provision of the multidisciplinary foot care team has probably resulted in relatively high numbers of amputations. The cost of diabetic foot care remains disproportionately high therefore as a result of these costly interventions. There is evidence from Marion Kerr’s report that the successful implementation of a multidisciplinary foot care team with podiatrists at the centre can lead to significant savings (28). For example, a study in Southampton demonstrated that a diabetic foot protection team working across primary and secondary care could reduce lengths of stay for foot ulcer admissions, as well as reducing major amputations [34]. Over a three year period, the median length of stay for foot ulcer admissions fell from 47 to 19 days. This resulted in an estimated saving of £1.3 million. The savings accrued from a reduction in major amputations.

## Conclusion

The provision of NHS diabetic foot services in terms of Foot Protection Teams and Multidisciplinary Foot Care Teams is poor in England. The National Inpatient Audit for 2011 demonstrated that 40.5% of the sites that were audited did not have a multidisciplinary foot care team. In many parts of the country there are no clear pathways for referral of increased-risk or high risk patients to foot protection teams, or for rapid referral of patients with new ulcers to MDTs. This is despite the recommendations from NICE. Quality standard 10 states that people with diabetes should be reviewed regularly by a foot protection team and that those presenting with an urgent foot problem should be seen and treated within 24 hours by a MDT. This standard clearly cannot be met by many hospital sites across England and Wales. NHS Diabetes Foot Care Network has appointed clinical and podiatry champions across England to try and drive improvements in service. In addition a joint campaign, ‘Putting Feet First’ run by Diabetes UK, NHS Diabetes and the Society of Chiropodists and Podiatrists is attempting to achieve improvements in diabetes foot services and reduce the unacceptably high rate of preventable amputations. When consideration is given to the cost efficiencies that have to be achieved by the NHS and the increase in prevalence of diabetes, improvements have to be made. It is time to put feet first.

## Competing interests

A McInnes represents the Society of Chiropodists and Podiatrists as a member of the ‘Putting Feet First’ campaign. He is also a NHS Diabetes Foot Care Network podiatry champion for the South East Coastal Region of England. In addition he is vice chairman of FDUK, a multidisciplinary group with an interest in diabetic foot disease. He is the editor-in-chief (education) for the Diabetic Foot Journal.
